# A parasitological survey and the molecular detection of *Entamoeba* species in pigs of East Java, Indonesia

**DOI:** 10.14202/vetworld.2023.650-656

**Published:** 2023-03-27

**Authors:** Dony Chrismanto, Nunuk Dyah Retno Lastuti, Makoto Matsubayashi, Lucia Tri Suwanti, Sri Pantja Madyawati, Dyah Ayu Kurniawati, Fransiska Cecilia Beka

**Affiliations:** 1 Program of Veterinary Science, Faculty of Veterinary Medicine, Universitas Airlangga, Surabaya, Indonesia; 2 Department of Health, Faculty of Vocational, Universitas Airlangga, Surabaya, Indonesia; 3 Study Program of Health Economics, Postgraduate School, Universitas Airlangga, Surabaya, Indonesia; 4 Department of Veterinary Parasitology, Faculty of Veterinary Medicine, Universitas Airlangga, Surabaya, Indonesia; 5 Department of Veterinary Science, Graduate School of Veterinary Science, Osaka Metropolitan University, Izumisano, Osaka, Japan; 6 Indonesian Research Center for Veterinary Sciences, Indonesian Agency for Agricultural Research and Development, Ministry of Agriculture Republic Indonesia, Indonesia; 7 Master Program, Faculty of Veterinary Medicine, Universitas Airlangga, Surabaya, Indonesia

**Keywords:** East Java, *Entamoeba*, Indonesia, pig, polymerase chain reaction, small-subunit ribosomal rRNA

## Abstract

**Background and Aim:**

In several countries, two *Entamoeba* porcine species, *Entamoeba suis* and *Entamoeba polecki* (subtype 1 and 3), have been detected using molecular methods and identified pathogenicity associated with enteritis. However, globally, *Entamoeba* infection prevalence in pigs is extremely limited. This study aimed to coprologically and genetically examine pig parasites to estimate prevalence of *Entamoeba* in three pig farms in East Java, Indonesia.

**Materials and Methods:**

Hundred porcine fecal samples (Landrace) were collected from three East Javan farms in well-known swine industry regions. Fecal samples were examined under a microscope after sugar-flotation centrifugation, and molecular species and subtype identification were performed using polymerase chain reaction (PCR) and primer pairs targeting small-subunit ribosomal RNA.

**Results:**

Microscopy examinations identified parasites in 89/100 fecal samples; *Entamoeba* spp. cysts were the most frequent in these samples. Polymerase chain reaction showed that 58 samples were comprised of mixed *Entamoeba suis* and *Entamoeba polecki*, 22 *E. suis* alone, and nine *E. polecki* alone infections. Epolec F6–Epolec R6 primers successfully amplified *E. polecki* ST1–4 subtypes, while Epolecki 1–Epolecki 2 amplified only the *E. polecki* ST1 subtype. *Entamoeba polecki* ST1-specific primers successfully detected the ST1 subtype in 19/67 *E. polecki* positive samples.

**Conclusion:**

*Entamoeba* spp. prevalence in Indonesian pigs was previously shown to be high. On coprological examination of East Javan pigs, we detected high *Entamoeba* spp. levels, in which we genetically identified as *E. suis* (80.0%), *E. polecki* (67.0%), and *E. polecki* ST1 (19%).

## Introduction

*Entamoeba* genus parasites are typically found in many vertebrate species, including humans and livestock [1], while several species, which infect pigs include *Entamoeba suis*, *Entamoeba polecki*, *Entamoeba histolytica*, and *Escherichia coli* [2–4]. The parasitic life cycle generally comprises two steps; trophozoites which represent motile and proliferative stages and cysts which represent environmental stages, but some species are excluded as they lack encysts [2, 3]. Cysts are shed in host feces and are resistant to disinfectants, thereby providing new host infection sources through oral routes. Conventionally, species classification within the genus was based on derived hosts and morphological data, such as zoites size or the number of nuclei in mature cysts [5]. Recently, to resolve difficulties related to morphological similarities, molecular analyses have been extensively used to distinguish species and genotypes [5–8]. In the genus, most *Entamoeba* spp. are believed to be harmless; however, some species such as *E. histolytica* in humans and animals, and *Entamoeba invadens* in reptiles, are highly virulent [1, 9].

To date, two *Entamoeba* spp., *E. suis* and *E. polecki*, have been reported in pigs [10, 11], although *E. histolytica* has been shown to infect mini pigs [12] experimentally. Several *Entamoeba* species exhibit zoonotic potential and include *E. polecki*, *E. histolytica*, and *E. coli* [3–5]. *Entamoeba suis* predominantly infects pigs, while *E. polecki* has been detected in multiple hosts, including humans and pigs. Sequence analysis of small-subunit ribosomal RNA (SSU rRNA) further subclassified *E. polecki* into four genetic subtypes (ST1–4) [5, 13]. ST1 was found in pigs and humans; ST3 in pigs, humans, and birds; and ST2 and ST4 in humans and primates [5]. Previously, the infection and disease characteristics of *E. suis* and *E. polecki* in pigs have not been reported. However, species/genotypes infecting pigs have been implicated in severe lesions associated with enteritis [7, 8]. *Entamoeba suis* invades the lamina propria and causes hemorrhagic colitis [14], while *E. polecki* induces refractory proliferative enteritis, which causes lethal lesions when combined with *Lawsonia intracellularis* or *Salmonella enterica* serovar Typhimurium [7, 8, 15, 16]. Although these reports come from Japanese pigs, it was reported that coinfection with *E. polecki* (unknown subtype) and *Brachyspira hyodysenteriae* was associated with necrotizing typhlocolitis in a Spanish pig with severe diarrhea [17]. Globally, *Entamoeba* infections in pigs are extremely limited and parasitic pathogenicity cannot be fully assessed based on the aforementioned data. However, a surveillance program in Tangerang, West Java, Indonesia, identified a high porcine *Entamoeba* spp. prevalence; 81.1% *E. suis*, and 18.4% and 17.3% *E. polecki* ST1 and ST3, respectively [4]. These findings potentially suggest that some Indonesian pigs are frequently infected with these parasites, although no other data are available.

This study aimed to coprologically and genetically examine pig parasites to estimate prevalence of *Entamoeba* in three pig farms in East Java, Indonesia. We used several molecular methods (polymerase chain reaction [PCR], sequencing, and phylogenetic tree analysis) using SSU rRNA as a marker. We also detected other coinfection gastrointestinal parasites.

## Materials and Methods

### Ethical approval

The research protocol was reviewed by our Local Animal Care and Use Committee (Ethics Clearance No. 1.KE.105.08.2021) under the guidance of the Ethical Clearance Commission Faculty of Veterinary Medicine, Universitas Airlangga, Indonesia.

### Study period and location

The study was conducted from November to January 2021. The samples were collected from three pig farms in East Java, Indonesia. The samples were processed at the Laboratory of Biomolecular, Faculty of Veterinary Medicine, Universitas Airlangga.

### Fecal sampling

Hundred porcine fecal samples (Landrace) were collected from three well-known swine industry regions in East Java; Farm A, Mojokerto; Farm B, Malang; and Farm C, Tulungagung. These farms were selected as they were the largest managed farms in each area (approximately 6000 pigs at Farm A, 10,600 at Farm B, and 14,400 at Farm C). At Farm A, 68 fecal samples (from 3 to 6-month-old animals) were collected immediately after defecation at a slaughterhouse. Fecal samples were randomly collected at the other farms: Farm B; 4 from <3-month-old, 7 from 3 to 6-month-old, and 5 from >6-month-old animals; and at Farm C, 1 from <3-month-old, 12 from 3 to 6-month-old, and 3 from >6-month-old animals. At all farms, 10–20 post weaned piglets were housed in pig pens on sawdust or soil floors until 3 months old. Pigs > 3 months old were reared individually in cages. Pigs were treated monthly for nematodes using anthelmintics such as albendazole and mebendazole. Animals showed no clinical symptoms, including diarrhea during sampling (91 normal and nine soft feces samples). Pig ages during sampling were recorded. Feces were stored in plastic bags at 4°C until examination.

### Parasitological examination

Fecal samples were examined under microscopy after sugar-flotation centrifugation according to a modified method [18–20]. Briefly, 5–10 g fecal sample was diluted in distilled water and filtered through gauze. After centrifugation, a sugar solution (specific gravity = 1.2) was added to the sediment and the samples were centrifuged. Parasites floating on the sugar solution surface were recovered using a Pasteur pipette and washed in distilled water. Finally, purified parasites were resuspended in 1 mL phosphate-buffered saline and stored at 4°C. Next, a 15 μL aliquot of parasite solution was placed onto a glass slide and smears were examined under a light microscope (Olympus, Japan) to enumerate parasites [20, 21]. Samples positive for *Entamoeba* cysts underwent molecular analyses.

### Molecular identification of Entamoeba spp.

*Entamoeba* spp. were identified using purified parasite aliquots (500 μL). After centrifugation, 0.5–0.7 mL DNAzol^®^ (Molecular Research Center, OH, USA) was added to sediments, samples were subjected to three freeze–thaw cycles to disrupt cysts, and samples were further processed according to the DNAzol protocol.

For species and subtype identification, PCR was performed using primer pairs targeting SSU rRNA (Table-1) [6, 7, 22, 23]. Specifically, 764-RD3 and 764–765 primers were used in nested PCR reactions and yielded approximately 320 bp *E. suis* fragments [22]. Epolec F6–Epolec R6 primers were used to generate ~430 bp *E*. *polecki* ST1–4 fragments [7]. Furthermore, Epolecki 1–Epolecki 2 were used to amplify approximate 200-bp *E. polecki* ST1 fragments [6]. In addition, EntaF-EhR primers were used to screen for *E. histolytica* and generated amplicons of approximately 170 bp [23]. An amplification reaction volume was 25 μL. The reaction mixture contained of 2 mL DNA template, 1 μL of each primer, 8.5 μL distilled water, and 12.5 μL Master mix (Bioline, Taiwan). Amplicons were separated using 1.5% agarose gel electrophoresis (Nacalai Tesque, Kyoto, Japan), stained with Gel Stain (GreenStar™ Nucleic Acid Staining Solution I, Bioneer, Daejeon, Korea), and visualized using a UV transilluminator.

**Table-1:** Specific primers used to detect *Entamoeba* species.

Species	The sequence of primers (5′–3′)	Amplicon size (bp)	Reference
*E. polecki* ST1	Epolecki 1 TCG ATA TTT ATA TTG ATT CAAATG Epolecki 2 CCT TTC TCC TTT TTT TAT ATT AG	210	[6]
*E. polecki*	Epolec F6 AAA TTA CCC ACT TTT AAT TTA GAG AGG Epolec R6 TTT ATC CAA AAT CGA TCA TGA ATT TT	430	[7]
*E. suis*	F-ATC AAA TCA ATT AGG CAT AAC TA R-AAT TAA AAC CTT ACG GCT TTA AA	320	[22]
*E. histolytica*	EntaF ATG CAC GAG AGC GAA AGC AT EhR GAT CTA GAA ACA ATG CTT CTC T	170	[23]

*E. polecki=Entamoeba polecki*, *E. suis=Entamoeba suis*, *E. histolytica=Entamoeba histolytica*

### Bioinformatics

To confirm sequencing outputs, the *E. polecki* amplicon from a Mojokerto sample (MJK_B28) was purified using a QIAquick PCR Purification Kit (Qiagen, Germany) and subjected to double-directional sequencing using PCR forward and reverse primers of *E. polecki* ST1 on an ABI PRISM 310 Genetic Analyzer (Applied Biosystems, USA). Sequencing was verified by inverting sequencing results from the reverse primer and aligning them to the forward primer sequence in the Clone Manager Professional 9 program (Version 9 for Windows, Scientific and Educational Software; http://www.scied.com) and comparing with a GenBank reference sequence (*E. polecki* pig, accession No. LC230018).

To identify closely related DNA sequences, the MJK_B28 sequence was used as a query in a National Center for Biotechnology Information nucleotide BLAST search; (http://www.ncbi.nlm.nih.gov>blast). The MJK_B28 sequence and all query hits with identities of >90% to the original (MJK_B28 sequence) were included in the dataset. Dataset sequences were then multiply aligned in ClustalW2 (http://www.ebi.ac.uk) [24] and a phylogram was generated using the neighbor-joining method [25] in MEGA6 [26].

## Results

**Figure-1:**
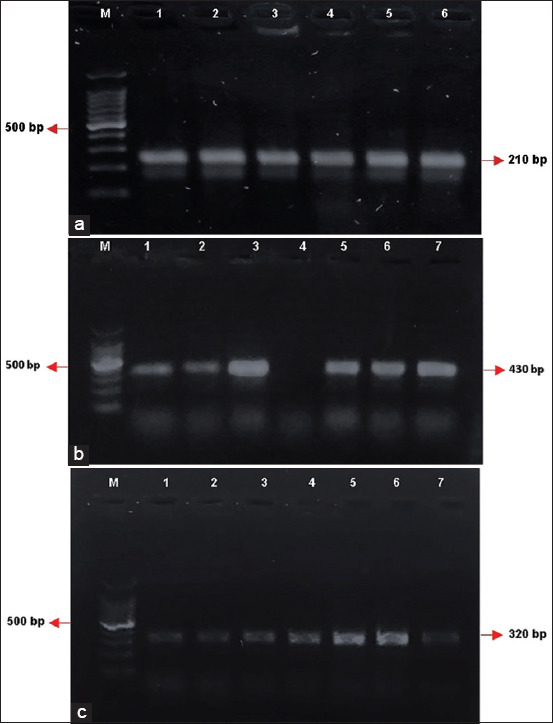
(a) Polymerase chain reaction gel showing *E. polecki* ST1 (210 bp) amplicons in East Javan pigs. M = DNA ladder. Lanes 1–6 show positive samples. (b) Polymerase chain reaction gel showing *E. polecki* universal (430 bp) in East Javan pigs. M = DNA ladder. Lanes 1, 2, 3, 5, 6, and 7 are positive samples and lane 4 is a negative sample. (c) Polymerase chain reaction gel showing *Entamoeba suis* (320 bp) amplicons in East Javan pigs. M = DNA ladder. Lanes 1–7 show positive samples. *E. polecki*=*Entamoeba polecki*.

Microscopy examinations identified parasites in 89/100 fecal samples (Table-2). *Entamoeba* spp. cysts were the most frequently observed cysts, although samples may have included other organisms similar to cysts as iodine staining was not performed. The average number of cysts ranged from approximately 10^2^–10^3^, and age-dependent tendencies could not be determined for Farm B and C animals. The polymerase chain reaction of the 89 samples showed that 58 had mixed *E. suis* and *E. polecki*, 22 with *E. suis* only, and nine with *E. polecki* infections only. Epolec F6–Epolec R6 primers successfully amplified *E. polecki* ST1–4 subtypes, while Epolecki 1–Epolecki 2 primers successfully amplified only the *E. polecki* ST1 subtype. *Entamoeba polecki* ST1-specific primers identified the ST1 subtype in 19/67 *E. polecki* positive samples. Because two *E. polecki* subtypes (ST1 and ST3) were previously identified in pigs, there was a possibility that the 67 *E. polecki* positive samples contained *E. polecki* ST3. *Entamoeba histolytica* detection was negative. Polymerase chain reaction amplicons from *E. suis*, *E. polecki*, and *E. polecki* ST1 are shown (Figures-1a–c).

**Table-2:** Study areas and *Entamoeba* spp. and gastrointestinal parasite results.

Farm	Subdistrict	Animal age (months)	No. of animals	Microscopy observations*				PCR analyses	
	
				*Entamoeba* spp.	*Eimeria* spp./*Cystoisospora suis*	*Trichuris suis*	*Ascaris suum*	*E. suis*	E. polecki** (ST1)
A.	Mojokerto	3–6	68	57 (479.1; 66–1,320)	20 (316.8; 66–1,452)	10 (316.8; 66–792)	10 (178.2; 66–330)	52	47 (19)
B.	Malang	<3 3–6 >6	4 7 5	4 (2442.0; 396–6,204) 7 (1821.6; 660–3,630) 5 (1942.3; 726–4,620)	1 (6,042) 0 1 (1,584)	0 1 (262) 3 (1,496.0; 264–3,234)	1 (462) 0 0	3 6 4	4 (0) 5 (0) 2 (0)
C.	Tulungagung	<3 3–6 >6	1 12 3	1 (264) 12 (671.0; 132–2,046) 3 (924.0; 396–1,980)	0 2 (165.0; 132–198) 0	1 (66) 5 (330.0; 66–924) 1 (1,518)	0 0 0	1 11 3	1 (0) 6 (0) 2 (0)
Total (%)			100	89	24	21	11	80	67 (19)

* Parentheses indicate the average number and range of parasites in 1 g feces.

** *E. polecki* was identified using universal primers for ST1–4. Parentheses show the number of ST1 subtypes identified using ST1-specific primers in *E. polecki* positive samples. *E. suis=Entamoeba suis*, *E. polecki=Entamoeba polecki*, PCR=Polymerase chain reaction

**Figure-2:**
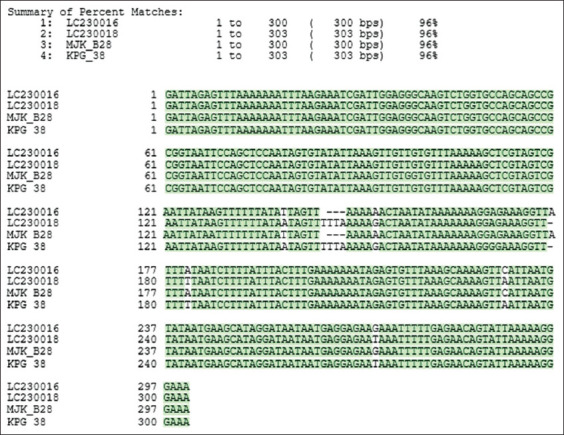
Small-subunit ribosomal RNA sequence alignments in *Entamoeba polecki* Mojokerto (MJK_B28) and comparisons with GenBank data (LC230016 and LC230018).

**Figure-3:**
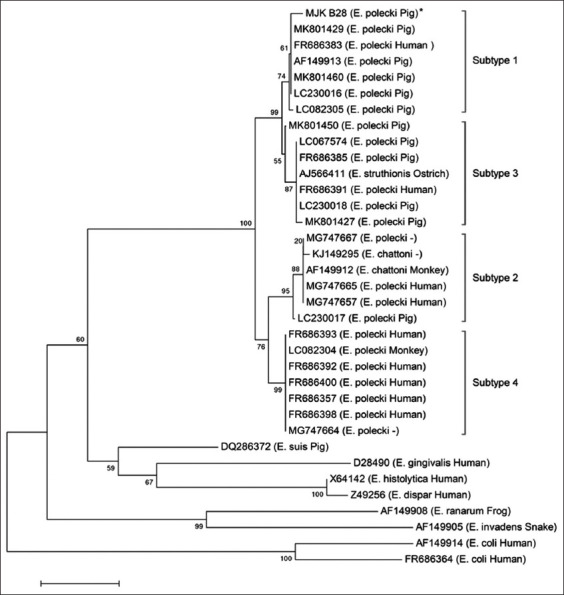
Phylogenetic analysis of partial 18S ribosomal RNA genes from different *Entamoeba* species. The partial *Entamoeba polecki* gene from a pig in Mojokerto (MJK_B28) was compared with corresponding genes in closely related *Entamoeba* spp. (Retrieved from the National Center for Biotechnology Information nucleotide database – accession numbers are indicated). Several related sequences, but with significantly less identity, were used as outgroups. Sequences were multiply aligned in ClustalW2 [24]. The phylogram was assembled using the neighbor-joining method [25]. *Entamoeba* spp. subtypes are indicated and bootstrap values from 1000 replications are shown for each branch. The scale bar indicates a phylogenetic distance of 0.05 nucleotide substitutions/site.

*Entamoeba polecki* Mojokerto (MJK_B28) alignment to gene bank sequence data (LC230016 and LC230018 accession numbers) showed 96% identity (Figure-2). Phylogenetic tree analysis showed that *E. polecki* from Mojokerto (MJK_B28) was identified in the *E. polecki* ST1 group and was relatively close to *E. polecki* in humans (FR686383) and *E. polecki* in pigs from other countries (MK801429, AF149913, MK801460, LC230016, and LC082305 accession numbers). This analysis also used out groups (less closely related to the in-group) with several related sequences, such as several *Entamoeba* species in humans (*Entamoeba gingivalis*, *E. histolytica*, and *Entamoeba dispar*), *Entamoeba* *ranarum* (frog), *Entamoeba* *invader* (snake), and *E. coli* (human) (Figure-3) [24, 25].

## Discussion

This is the first report showing *Entamoeba* spp. cyst percentages in pig feces. Average and maximum numbers were not found to be differences among pig ages; however, a thorough statistical analysis was not performed due to low study animal numbers. The majority of feces samples were normal and pigs exhibited no clinical symptoms. During *E. histolytica* infection, cysts are generally found in the stool, while trophozoites are typically found in watery or dysenteric feces concomitant with clinical symptoms [27]. Furthermore, species-specific immunity during primary infection with *Entamoeba* spp., such as *E. histolytica*, may have key resistance roles against reinfection [28, 29]. In our study, cysts identified in formed stool, with no clinical signs in animals, may have reflected acquired immunity following the previous infections; however, further investigations with more samples, especially from younger animals (e.g., < 3 months old), are required to elucidate parasite pathogenicity, especially during initial infections.

To date, *Entamoeba* spp. have been detected in fecal specimens (ranging from a few to approximately 10) in several countries, for example, Indonesia, Sweden, the United Kingdom, and Germany [5, 10, 30]. Recently, in more than 500 pigs in China, *E. suis*, *E. polecki* ST1, and ST3 were identified by Li *et al*. [31] in 13.0%, 45.2%, and 34.1% of samples, respectively, while Ji *et al*. [3] observed that 0.8%, 38.2%, and 10.0% of samples were positive, respectively. In West Java, the prevalence of *E. suis*, *E. polecki* ST1, and ST3 in 196 fecal samples was 81.1%, and 18.4%, and 17.3%, respectively [4]. In our study, the Mojokerto amplicon, using species-specific *E. polecki* ST1 primers, showed 19% (19/100) positivity. The MJK_B28 sample was confirmed by sequencing and a phylogenetic tree identified 96% homology with *E. polecki* ST1 in humans (FR 686383 and LC 230016) (Figures-2 and 3). These are a new finding of mixed infection with *E. suis* and *E. polecki* ST1, and coinfection with *Eimeria* spp., *Isospora suis*, *Trichuris suis*, and *Ascaris suum* (Table-2), in Mojokerto, East Java, Indonesia. According to Stensvold *et al*. [5], *E. polecki* is a protozoan parasite in the digestive tract that attacks pigs, monkeys, primates, and birds and has zoonotic potential. Small-subunit ribosomal RNA sequence analysis resulted in the further subclassification of *E. polecki* into four genetic subtypes (ST1–4) [3, 4, 7]. ST1 is found in pigs and humans, the most frequently reported zoonoses are ST1 and ST3, while ST2 and ST4 are specific subtypes in humans and non-human primates [5]. Our results were consistent with this report and confirmed high *E. suis* prevalence in Indonesian pigs, although surveillance of more pigs over a wider geographical area is required in future studies.

Other parasites, including *Eimeria* spp. oocysts or *Cystoisospora suis*, were identified in 24 samples, while *A. suum* and *T. suis* eggs were identified in 11 and 21 samples, respectively. All samples contained mixed *Entamoeba* spp. infections. Although few reports focusing on gastrointestinal parasites in Indonesian pigs have been published, *Eimeria* spp. or *C. suis*, *T. suis*, and *A. suum* prevalence is reported as 42.2%, 7.8%, and 11.8% in healthy pigs, and 38.6%, 52.3%, and 9.1% in dead pigs, respectively, in Central Papua (no *Entamoeba* spp. descriptions are provided) [32]. In another West Javan study, *Eimeria* spp. or *C. suis*, *T. suis*, and *A. suum* prevalence was reported as 79.1%, 4.6%, and 1.0%, respectively [4, 33]. These findings suggest that coccidian prevalence, including *Eimeria* spp. or *C. suis*, is high in Indonesian pigs, with *E. suis* the most prevalent.

Gastrointestinal parasite infections are transmitted through fecal–oral routes and may be associated with farm management systems [34]. In our study, 10–20 piglets were reared in the same pen, suggesting that parasite transmission and initial infections had easily occurred. In addition, we conducted our study during the rainy season; therefore, parasite spread may have occurred more through contaminated rainwater. In future studies, we will compare parasite prevalence between rainy and dry seasons to understand the impact of different climate conditions. Because porcine *Entamoeba* spp. virulence remains largely uncharacterized, larger studies on younger or preweaned piglets are required to fully understand parasitic prevalence and clarify pathogenicity and associated effects on porcine productivity during breeding.

## Conclusion

*Entamoeba* spp. prevalence in Indonesian pigs is too high. We detected high *Entamoeba* spp. levels during coprological examinations in East Javan pigs. Parasites were genetically identified as *E. suis* (80.0%), *E. polecki* (67.0%), and *E. polecki* ST1 (19%). Previously, a high *E. suis* prevalence was reported in Indonesian pigs; therefore, our results are congruent with these findings, although our sample numbers were low. In addition, we detected *E. polecki* ST1, which is a potentially zoonotic protozoan. In future studies, more samples are required to evaluate parasite pathogenicity, especially during initial infection stages in younger animals.

## Authors’ Contributions

NDRL and LTS: Conceptualized and designed the study. DC, SPM, DAK, and FCB: Performed the research, collected and analyzed the data. NDRL and MM: Interpretation of the data, drafted and revised the manuscript. All authors have read, reviewed, and approved the final version of the manuscript.
